# Chitosan–Silica Composite Aerogel for the Adsorption of Cupric Ions

**DOI:** 10.3390/gels10030192

**Published:** 2024-03-11

**Authors:** João P. Vareda, Pedro M. C. Matias, José A. Paixão, Dina Murtinho, Artur J. M. Valente, Luisa Durães

**Affiliations:** 1 University of Coimbra, CERES, Department of Chemical Engineering, Rua Silvio Lima, 3030-790 Coimbra, Portugal; jvareda@eq.uc.pt (J.P.V.); luisa@eq.uc.pt (L.D.); 2 University of Coimbra, CQC-IMS, Department of Chemistry, Rua Larga, 3004-535 Coimbra, Portugal; petermatias1998@gmail.com (P.M.C.M.); dmurtinho@ci.uc.pt (D.M.); 3 University of Coimbra, CFisUC, Department of Physics, Rua Larga, 3004-516 Coimbra, Portugal; jap@fis.uc.pt

**Keywords:** chitosan, silica, aerogel, sorption, copper

## Abstract

A chitosan–silica hybrid aerogel was synthesized and presented as a potential adsorbent for the purification of cupric ion-contaminated media. The combination of the organic polymer (chitosan), which can be obtained from fishery wastes, with silica produced a mostly macroporous material with an average pore diameter of 33 µm. The obtained aerogel was extremely light (56 kg m^−3^), porous (96% porosity, 17 cm^3^ g^−1^ pore volume), and presented a Brunauer–Emmett–Teller surface area (*S*_BET_) of 2.05 m^2^ g^−1^. The effects of solution pH, aerogel and Cu(II) concentration, contact time, and counterion on cupric removal with the aerogel were studied. Results showed that the initial pH of the cation-containing aqueous solution had very little influence on the removal performance of this aerogel. According to Langmuir isotherm, this material can remove a maximum amount of ca. 40 mg of cupric ions per gram and the kinetic data showed that the surface reaction was the rate-limiting step and equilibrium was quickly reached (in less than one hour). Thus, the approach developed in this study enabled the recovery of waste for the preparation of a novel material, which can be efficiently reused in a new application, namely water remediation.

## 1. Introduction

Mankind is currently very concerned about environmental pollution, in particular that of water bodies and food, with heavy metals. Heavy metals refer to a group of chemical elements that are toxic to living organisms and have a density of more than 4 g cm^−3^ [1,2]. The United States Environmental Protection Agency (US EPA) recently released a survey on drinking water infrastructure, which estimated that 9.2 million lead services’ lines are still being used to distribute drinking water to citizens [3]. These pipes contaminate drinking water, slowly poisoning people. Additionally, Consumer Reports published a study on heavy metal concentration in baby foods and found relevant amounts of very toxic elements, which led them to consider most of the tested products as concerning [4]. Food products made from rice or sweet potato had higher levels of heavy metals compared to other products, which are attributed to the ability of these plants to absorb heavy metals from soil. These reports are good examples of how anthropogenic emissions can cause significant pollution and impact our health.

Copper is the third most important metal, measured by weight [5]. This heavy metal has a high yearly production [6,7] and is also a micronutrient for animals and plants [8]. We mainly use it in electrical equipment, but also in plumbing/construction, machinery, art, music instruments [5,9] and for catalysis [10,11,12]. It is worth mentioning that the increase in production of electric vehicles, along with other efforts towards green energy, has raised the demand for copper. Copper is a vital raw material, but it can also be a major source of man-made pollution [5,13,14]. In humans, copper can cause anemia, damages to the liver and kidneys, and stomach irritation [5,14]. Thus, this metal is very important for society as a raw material, but as a pollutant, it disrupts ecosystems and human well-being.

The adsorption process has been widely studied with the intent of purifying water from harmful substances [15]. In this work, we report a hybrid silica–chitosan aerogel for the sorption of cupric ions. Chitosan is a polymer derived from the biopolymer chitin, which is highly prevalent in fishery waste. Therefore, we promote the creation of value-added products from wastes, reducing their amount and contributing to a blue economy. On the other hand, silicas are also considered low-toxicity materials, known for their thermal stability, resistance in acidic and microbial environments, highly developed surface, and promising kinetics [16,17]. So, the novelty of this study is based on the construction of a new aerogel material from these two accessible and low-cost reagents (chitosan and silica), with the aim of limiting the use of starting substances of concern to obtain high-performance materials for the environmental remediation of copper-containing waters.

The strategy developed in this work is a synthetic opportunity, because although a plethora of materials have already been reported for the removal of Cu(II) [18], research into aerogels, as gels whose liquid part is replaced by a gas, and specifically into aerogels based on both chitosan and silica, is still very limited for this application field [19,20]. Furthermore, we describe a simpler aerogel synthesis procedure in a one-pot fashion that requires no additional stages likes silylation, and the chitosan and silica phases are covalently bonded using silica as the crosslinker, unlike other hybrids in the literature [21,22,23,24,25,26]. Therefore, an interesting combination between the key characteristics of aerogels as superior adsorbents (i.e., surface adjustability, low density, highly porous structure, and typically high surface area [19]) and the inherent properties of chitosan and silica may be achieved.

## 2. Results and Discussion

### 2.1. Properties of the Composite Aerogel Adsorbent

**Figure 1:**
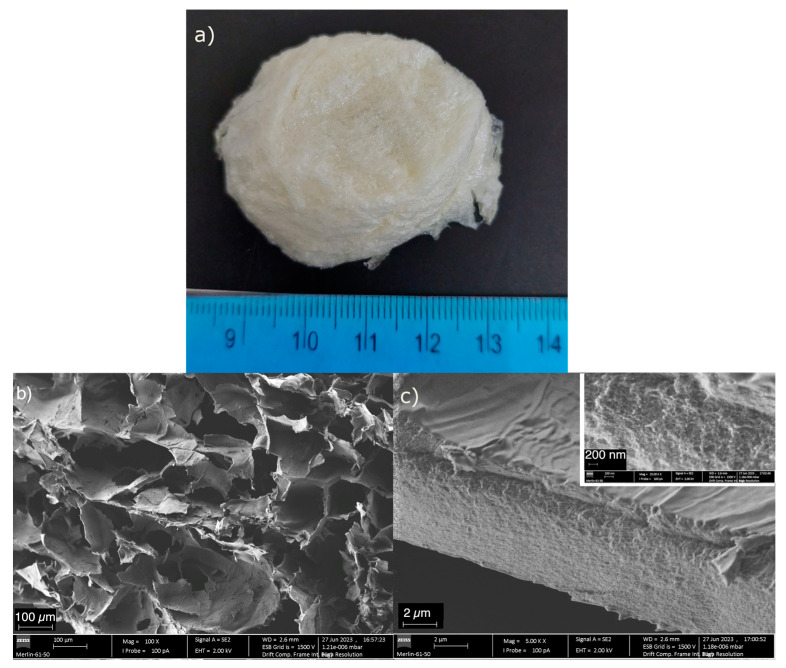
Aspect (**a**), and microstructure of the chitosan–silica aerogel at ×100 (**b**) and ×5 K (**c**) magnification. Inset detailing the sample’s microstructure across the height of the solid obtained at ×25 K magnification.

A photograph of the aerogel and its micrograph, at two different magnifications, are presented in Figure 1. The adsorbent features a sponge-like appearance (Figure 1a), a yellow tone, and is also easily compressible to the touch. The aged gel batches swelled when washed prior to freeze drying, which led to the loss of their cylindrical shape, obtained in the polypropylene mold where gelation and aging occurred. This composite is mostly macroporous (Figure 1b), and its microstructure is more akin to that of freeze-dried polymer hydrogel than to that of silica aerogel. This is surely due to the high amount of chitosan in the composite. The microstructure in Figure 1 is similar to that reported by Pandis et al. [21] and Zhu et al. [22]; these authors first created a chitosan scaffold, freeze dried it, and then incorporated silica via silylation in solution. On the other hand, it is dissimilar to other reported silica–chitosan gels [23,24,25,26], probably due to the synthesis conditions (amount of chitosan in the composite and silylation step). In Figure 1c, the silica network can be seen along the height of the solid portion. The porous structure is not the same in the whole material; the surface of the pores is very dense and does not feature many visible pores, maybe due to the compression of ice crystals’ growth during drying on the pore walls. However, the inset in this figure shows that the material inside the pore walls is still porous (fracture surface).

Despite chitosan and silica precursors being covalently bonded, an isotropic dispersion of both phases would not allow the silica network to grow since chitosan has a high molecular weight. Thus, the silica network acts as a crosslinker to chitosan. This can possibly lead to exfoliated layers, as seen in Figure 1c.

The aerogel composite is very light, even for aerogel standards [27], and shows extreme porosity—Table 1. Because of its macroporous nature, as observed in Figure 1b, the specific surface area is very reduced, and the average pore is micron sized, much larger than those obtained with other silica aerogel composites [28,29]. The porosity disclosed in the inset of Figure 1c is not likely to have been measured using the gas adsorption technique, as it is covered by an almost non-porous surface, and is only revealed in fractured surfaces. When compared to other silica–chitosan aerogels, the one reported here has a much lower specific surface area, but a higher pore volume and average pore size [26,30,31,32]. 

**Figure 2:**
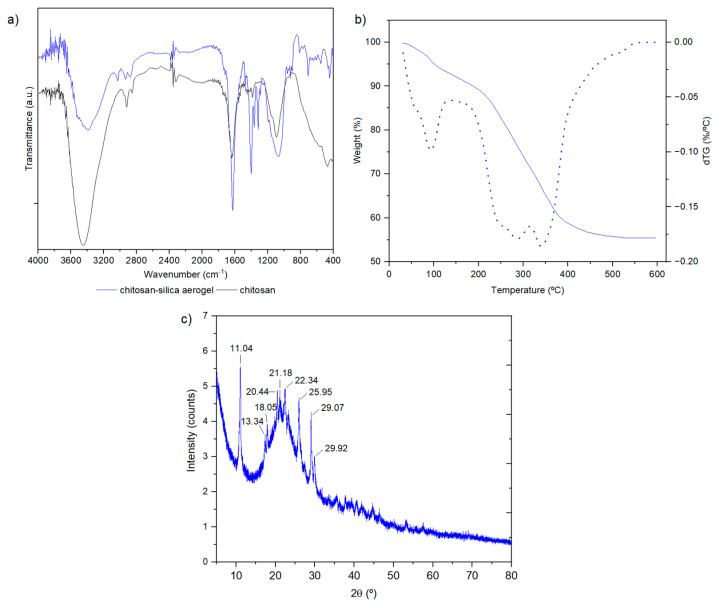
FTIR spectra of chitosan–silica aerogel (solid blue line) and chitosan (solid black line) (**a**); thermogram (weight, %, solid blue line) and respective derivative (dTG, dotted blue line) (**b**); and XRD pattern (**c**) of the chitosan–silica aerogel.

Regarding the chemical characterization of the composite, the Fourier-transform infrared (FTIR) spectra of both chitosan and the aerogel are shown in Figure 2a, and the elemental analysis results are reported in Table 2. The thermogravimetric analysis and X-ray diffraction (XRD) pattern of the aerogel are presented in Figure 2b and Figure 2c, respectively.

The infrared spectra of chitosan and chitosan–silica aerogel are very similar. However, the introduction of the silica matrix and ethylenediaminetetraacetic acid (EDTA) brings some noteworthy changes: a new band at 3030 cm^−1^ can be ascribed to the C-H stretching vibration of the α-carbon in the carboxylic group of EDTA [33,34]; shoulders at ~1730–1700 cm^−1^ indicate the presence of the carboxylic acid groups (carbonyl stretching vibration); and bands in the 1400–1300 cm^−1^ region, which correspond to the bending of methylene groups, become more intense because the silica phase also features these groups. Additionally, in the 900–400 cm^−1^ region, the spectrum of the composite features multiple bands due to the bending and symmetric stretching vibrations of siloxane bonds, as well as the stretching of Si-C bonds [35]. Considering the amount of EDTA used in the synthesis and the relative intensity of the bands in the FTIR spectrum, it seems that only a fraction of this molecule is retained in the aerogel. However, a quantitative analysis of this spectrum, given the multiple overlaps of bands, is not possible. The stretching of the C-O-C (~1150 cm^−1^) and C-O (~1090 cm^−1^) bonds [36] in the chitosan spectrum overlaps with the two modes of the asymmetric stretching vibration of Si-O-Si bond, creating two shoulders at ~1190 and 1150 cm^−1^, and a broad band at ~1070 cm^−1^ in the spectrum of the composite aerogel.

The CHNS content of the aerogel reveals that It contains approximately 3.5 wt% of nitrogen atoms, which come from both chitosan and EDTA, since the silica-based structure does not have this element. Considering the results in Figure 2a, it might be assumed that most of the nitrogen come from chitosan. The amount of sulfur in the sample is consistent with the expected value; if the sample was composed by only the silica-based structure, this element would contribute to 7.7 wt%, but the mass amount of chitosan is near 50 wt% (and not accounting for the EDTA added to the mixture), and thus the final percentage of S should be less than half of the given value for only organically modified silica. This confirms the presence of (3-mercaptopropyl)trimethoxysilane (MPTMS) in the silica network.

The thermogravimetric curve plotted in Figure 2b reveals that the aerogel has two thermal-degradation phenomena that overlap. The first occurs from approximately 30 to 134 °C, with a mass loss of 6.8 wt%, and can be associated with adsorbed water. The following phenomena occurs from 134 to 550 °C, being associated with the degradation of the organic moieties in the sample, and results in a mass loss of 37.5 wt%.

In the X-ray diffraction pattern of the aerogel (Figure 2c), nine crystalline reflections were found for 2θ between 11 and 30°, at 11.04, 17.34, 18.05, 20.44, 21.18, 22.34, 25.95, 29.07, and 29.92°. The broad band at 2θ = 18–30° is characteristic of the bond distances of the short-range order in chitosan, possibly of semi-crystalline structure, since according to the literature, it is possible to assign the values of 2θ = 11.04, 17.34/18.05, 20.44/21.18, 22.34, and 25.95° to the reflection planes 020, 110, 120, 101, and 130 of chitin, respectively [37]. This result shows not only that the starting chitosan is not completely deacetylated, but also that its degree of deacetylation is high (>75%), as the 020 reflection is considerably shifted towards a higher diffraction angle (11.04°) compared to that observed for chitin (9.39°) [37,38,39,40]. Less-intense diffraction peaks near 2θ = 30° can also identify the presence of chitosan in the aerogel structure, as observed by Jia et al. [41]. 

### 2.2. Influence of Test Parameters on the Adsorption of Cupric Ions

**Figure 3:**
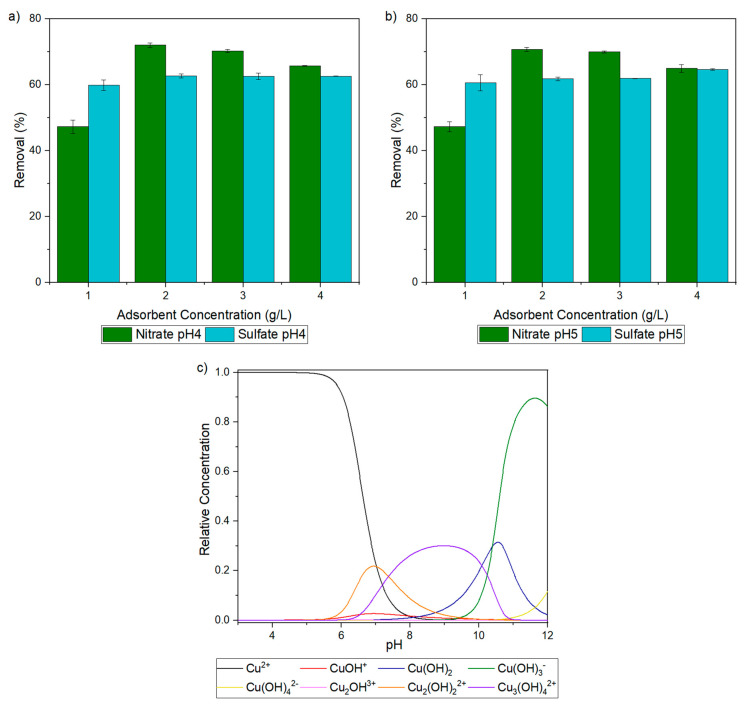
Cupric ion removal with the chitosan–silica aerogel from nitrate and sulfate salt solutions at pH = 4 (**a**) and pH = 5 (**b**), and cupric ions speciation diagram in the Cu^2+^-OH system (**c**).

The effects of pH and adsorbent concentration on copper(II) adsorption are presented in Figure 3a,b for two different copper salts. pH values of 4 and 5 were selected because highly acid solutions (pH < 4) inhibit metallic cation sorption due to competition with hydronium ions’ adsorption [42,43], and, at these conditions, cupric ions are free in solution, as shown in the speciation diagram of Figure 3c. This diagram was constructed with the data provided in Powell Kipton et al. [44], considering a total cupric concentration of 1 mM, and shows that at pH > 6.5, the precipitation of copper in the form of Cu(OH)_2_ becomes relevant, which will also compete and negatively affect the removal of metals with the aerogel.

Because the cupric ions are in a free state in both tested pH values, for this adsorbent, the removal performance for each salt is virtually unchanged from pH = 4 to pH = 5. For an aerogel concentration of 1 g/L, the removal is always significantly smaller for copper(II) nitrate than for copper(II) sulfate. In fact, the lowest removal percentage presented in Figure 3 is verified at these conditions. However, the removal of copper(II) starting from the copper nitrate salt increases at the remainder concentrations, peaking at an aerogel dose of 2 g/L.

On the other hand, for copper(II) sulfate, the removal performance of Cu(II) can be considered constant with increasing aerogel concentrations, as there is just marginal improvement at the highest concentration. With the exception of the before-mentioned situation, verified at an aerogel concentration of 1 g/L, cupric removal is higher in copper(II) nitrate solutions than in copper(II) sulfate solutions, by as much as 10%. These results are different than those found in the literature [45], e.g., with primary amine modified silicas, for which copper(II) is less sorbed using copper nitrate than copper sulfate [45], and lower pHs have a significant negative effect on copper sorption [46].

Considering the obtained results, for the tests presented in Section 2.3 and Section 2.4, the applied adsorbent dose is 2 g L^−1^ and the initial solution pH is 4.

### 2.3. Adsorption Kinetics

**Figure 4:**
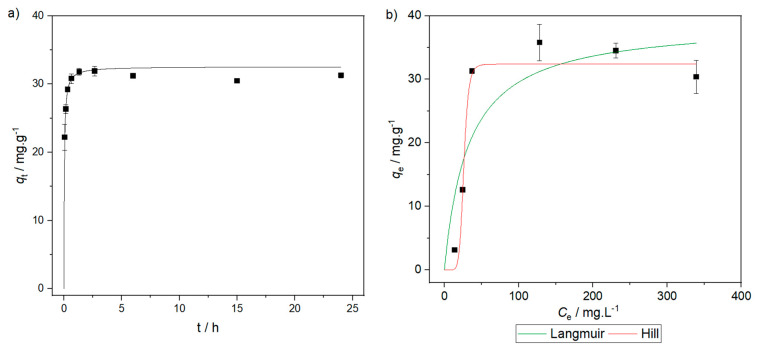
Kinetics (**a**) and isotherms (**b**) of cupric sorption. For the kinetics, a starting copper concentration of 100 mg L^−1^ was used.

The kinetic data for copper sorption, alongside the best model are plotted in Figure 4a. The parameters of the kinetic models fitted to the dataset are in Table 3.

The uptake of copper is very fast in the beginning, with more than half of the equilibrium removal being achieved in the first few minutes (first datapoint in Figure 4a), and equilibrium being reached in less than one hour. The goodness of fit criteria in Table 3 demonstrates that the pseudo-second-order model fitted to the data much better than the remainder, suggesting that adsorption is limited by the surface reaction. To further support this claim, given the ability of this kinetic model to fit to many distinct systems [47], the Weber–Morris model, based on Fick’s second law of diffusion and describing intraparticle diffusion [48], was also fitted to the data. The fitting results (Table 3) clearly show that the model does not fit to the data, as it has a low coefficient of determination (*R*^2^); hence, this system is not limited by intraparticle diffusion. The macroporous nature of the adsorbent facilitates diffusion through its structure, so a diffusion limited process would not be expected.

### 2.4. Adsorption Isotherms

The cupric sorption isotherms are plotted in Figure 4b. Only the isotherm models that are discussed below are represented. The fitting results for the isotherm models are shown in Table 4.

Two and three fitting parameter models have been fitted to the experimental data: Langmuir and Freundlich, and Hill isotherms. For the former models, the Langmuir equation is the one that better fits the sorption of cupric cations onto the aerogel. The Langmuir model describes adsorption at specific surface sites, which is consistent with the aforementioned interpretation that this sorptive process is controlled by the surface reaction. On the other hand, by applying the Hill equation, the lower-concentration data are better described, and it can also be concluded that the number of species sorbed per site (*n*) is equal to eight. From the plot, one can see that the dataset tends to plateau, despite the decrease in the ordinate coordinate in the last datapoint. This plateau is also achieved at relatively low concentrations, indicating that the material became saturated.

The equilibrium values of (39 ± 9) and (32 ± 2) mg g^−1^, obtained using Equations (8) and (10) (in Section 4.5), respectively, are also similar or better than the equilibrium uptake obtained for copper with different adsorbents, such as natural materials [18], in particular many chitosan-based sorbents and composites [49], and activated carbons [50], carbon nanotubes [51], some silica-based structures [52,53] and other aerogels [20,54]. On the other hand, the maximum adsorption capacity obtained for the synthesized aerogel is lower than that obtained for reduced chitosan [55] as well as for other adsorbents, ranging from simple, bio-based materials, to purely synthetic materials of greater structural complexity (see Table 5). However, in most reported cases, it should be noticed that materials cannot be obtained as monoliths, which limits their potential for recuperation and recycling, and their removal kinetics are often slower, which is not the case with the synthesized chitosan–silica composite aerogel, as it is generally superior regarding the process scalability and quickness.

### 2.5. Effect of Cupric Ion Adsorption on Aerogel Structure

The morphology and surface chemical composition of the aerogel before and after copper(II) adsorption were compared in order to evaluate structural differences after the adsorption process and prove the efficiency of copper(II) removal.

**Figure 5:**
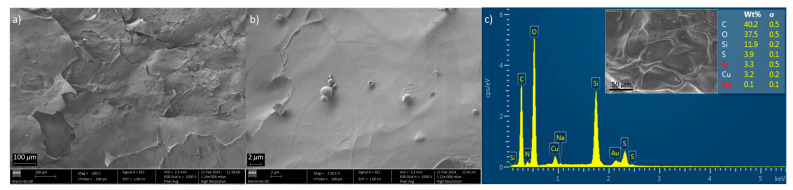
Microstructure at 100× (**a**) and 2.5 K× (**b**), and EDX spectrum (**c**) of the copper-loaded chitosan–silica aerogel.

Comparing the SEM images in Figure 1b,c of the native aerogel with those of the aerogel after copper(II) uptake (Figure 5a,b), it can be seen that there is a porosity reduction and a more continuous surface after Cu(II) adsorption, possibly due to metal–aerogel interactions with the consequent pore filling and the effect of the material drying after adsorption. A sheet-like surface characteristic of the neat aerogel (Figure 1b) is also observed after adsorption (Figure 5a). Additionally, the copper-loaded aerogel shows the presence of spherical aggregates (Figure 5b), in which, according to the respective energy-dispersive X-ray (EDX) spectra (see Figure S1), there is a higher amount of copper (8–12 wt%) compared to the remaining structure, where the surface is smoother, and the copper content is 3–4 wt%. These spherical particles may result from the precipitation of copper(II) salts on the surface after aerogel freeze-drying. The combination of EDX spectroscopy (Figure S1) and backscattered electrons on SEM imaging (Figure S2) made it possible to identify that the brightest zones of the mapping of Figure S2 coincide with spherical locations where the greatest accumulation of copper is found.

For the starting aerogel, the EDX spectrum (Figure S3) allowed the conclusion that C and O predominate in the structure containing chitosan and organically modified silica. The N content is due to the chitosan and EDTA, while the S percentage is due to the presence of MPTMS groups in the silica network, as described for CHNS analysis (Table 2). The incorporation of silica was also proved by the Si content of around 6 wt%, while the sodium content of 5–6 wt% in the structure derives from the use of EDTA in the form of a disodium salt. As the initial aerogel has no copper in its structure, the determination of copper on the post-adsorption aerogel surface using SEM/EDX (Figure 5c) confirmed the capacity of the composite material as a cupric ion adsorbent.

## 3. Conclusions

A hybrid aerogel composed of silica and chitosan was synthesized, characterized, and used as an adsorbent to remediate copper(II)-contaminated aqueous environments. Copper is present in the wastewater of many companies, despite being a metal of high commercial value. The composite aerogel presented a lightweight spongy monolithic structure, with a bulk density of 56 kg m^−3^, a specific surface area of 2 m^2^ g^−1^, 96% porosity and pore volume and average size of 17 cm^3^ g^−1^ and 33 µm, respectively. The mostly macroporous nature of the material resulted in a low surface area; however, a detailed observation of its microstructure revealed that micro and mesoporosity are still present. When tested as an adsorbent for copper(II) ion removal, the aerogel showed a maximum adsorption capacity of approximately 40 mg g^−1^ (Langmuir capacity), which was very similar to that obtained using other silica-based aerogels. Combining isotherm and kinetic analysis, the surface reaction was found to be the limiting step of the adsorption process, since the large pore size appears to facilitate the diffusion of the ions within the microstructure. Overall, the use of a very-low-cost and non-toxic polysaccharide has proved to be an excellent solution to give waste a new application and to reduce costs and increase the potential for the scale-up production of aerogels, while maintaining or increasing their adsorption efficiency, due to the functional groups it adds to the aerogel structure (e.g., -NH_2_ and -OH).

## 4. Materials and Methods

### 4.1. Materials

Tetraethyl orthosilicate (TEOS, 98%), (3-mercaptopropyl)trimethoxysilane (MPTMS, 95%), (3-glycidyloxypropyl)trimethoxysilane (GLYMO, ≥98%), chitosan of low molecular weight (deacetylation degree > 75%; 50,000–190,000 Da), anhydrous oxalic acid (≥99%), copper(II) sulfate pentahydrate (≥98.0%), and sodium hydroxide (≥98%, pellets, anhydrous) were purchased from Sigma-Aldrich (Darmstadt, Germany). Ethylenediaminetetraacetic acid disodium salt dihydrate (EDTA, 99+%) and nitric acid (65%) were supplied from Fisher. Ethanol (EtOH, ≥99%) was bought from Valente e Ribeiro, while copper(II) nitrate hemi(pentahydrate) (p.a.) was purchased from Chem-Lab (Zedelgem, Belgium). All substances were used as received. Milli-Q water was used whenever needed.

### 4.2. Synthesis of the Chitosan–Silica Composite Aerogel

First, 0.3 g of chitosan was dissolved in 15 mL of an aqueous solution of acetic acid (2% *v*/*v*) for 90 min at 60 °C, in a closed polypropylene container, under stirring. Then, 0.2 mL of GLYMO and 0.315 g of EDTA were added, under stirring, and the mixture was left to react for 24 h at 60 °C. Meanwhile, in a glass beaker, 0.4 mL of TEOS and 0.2 mL of MPTMS were diluted in 5 mL of ethanol and hydrolyzed with 0.1 mL of a 0.01 M oxalic acid aqueous solution at 27 °C. After 30 min of stirring, this solution was left in an oven for a day. After 24 h, the hydrolyzed silica precursors were mixed with the chitosan solution, under stirring at 60 °C. The resulting solution was stirred for 30 min before it was placed in an oven to gel and age for a day at 50 °C. The solvent of the solution was composed of 25% ethanol and 75% of the acetic acid solution; its chitosan concentration was 15 g L^−1^. The molar ratios of EDTA:MPTMS:GLYMO:chitosan:TEOS were 0.5:0.5:0.5:1:1.

The resulting aged composite gels were demolded from the polypropylene container, washed with a liter of distilled water, frozen at −80 °C, and freeze dried for two days on an FDL-10N-80-TD-MM from MRC (Harlow, UK). 

### 4.3. Characterization

The characterization routines of aerogels are as follows: The bulk density (*ρ*_b_) was obtained by weighting the samples and measuring their dimensions on the three axes. The skeletal density (*ρ*_s_) of milled samples was assessed using He pycnometry (Accupyc 1330, Micrometrics, Norcross, GA, USA). The BET specific surface area (*S*_BET_) was obtained through nitrogen adsorption at 77 K (ASAP 2000, Micrometrics). Porosity, pore volume (*V*_pore_), and average pore size (*D*_pore_) were calculated in accordance with Equations (1)–(3). The composite’s microstructure was observed with a field-emission scanning electron microscopy (FE-SEM) (Merlin Compact/VPCompact FESEM, Carl Zeiss Microscopy GmbH, Jena, Germany). The surface chemical composition analyses using EDX were carried out on the previously described FE-SEM, equipped with an EDX spectrometer (SEM/EDX) (X-Max^N^ Silicon Drift EDX Detector, Oxford Instruments, Abingdon, UK). FTIR spectra (FT/IR 4200, Jasco, Mary’s Court Easton, MD, USA) were obtained with KBr pellets in the wavenumber range of 4000 to 400 cm^−1^, with 128 scans and a resolution of 4 cm^−1^. The CHNS content of powdered samples was determined using an elemental analyzer (EA Flash 2000, Fisher Scientific, Hampton, NH, USA). A thermogravimetric analysis (TGA) was carried out on a TG209 F3 Tarsus thermogravimetric analyzer (Netzsch Instruments, Burlington, MA, USA), where samples (ca. 3 mg) were heated under nitrogen from 25 to 600 °C, at 10 °C min^−1^, with a flow rate of 20 mL min^−1^. The XRD pattern was collected on a D8 Advance diffractometer (Bruker, Karlsruhe, Germany) equipped with a 1D LynxEye detector, using Ni-filtered Cu Kα radiation. The powder sample was mounted in a low-background off-cut silicon crystal sample holder. The X-ray diffraction pattern was collected using Bragg–Brentano geometry, at room temperature, by scanning in the angle range 5° ≤ 2θ ≤ 80° with a step of 0.01° and a dwell of 1 s per step.

(1) $$Porosity\left(\%\right)=\left(1-\frac{\rho_{b}}{\rho_{s}}\right)\times 100$$

(2) $$V_{pore}=\frac{1}{\rho_{b}}-\frac{1}{\rho_{s}}$$

(3) $$D_{pore}=\frac{{4V}_{pore}}{S_{BET}}$$

### 4.4. Batch Adsorption Tests

The chitosan–silica composite aerogel was first milled into coarse flakes. The adsorption performance was evaluated by mixing the adsorbent and the cation solution in a test flask and shaking it in a rotational stirrer (speed setting 16, REAX 20, Heidolph Instruments, Nuremberg, Germany) at 20 °C. A different flask was prepared for each datapoint and replica reported, ensuring that all tests were conducted independently. When the test ended, the solution was filtered using a 0.45 µm polytetrafluoroethylene (PTFE) syringe filter and stored at 4 °C until analysis. The concentration of copper in the filtrate was determined using flame atomic absorption spectroscopy, with an acetylene-air flame (Solaar 939 AAS, Unicam, Camerino, Italy).

The effects of counterion (sulfate and nitrate), adsorbent concentration (1, 2, 3 and 4 g L^−1^), and pH (4, 5) on the adsorption performance were studied in a 24 h equilibrium test with a copper solution of 100 mg L^−1^.

Kinetic tests were conducted with a starting copper concentration of 100 mg L^−1^, with an adsorbent dose of 2 g L^−1^ at pH = 4, with contact times ranging from 5 min to 24 h. Isotherm studies were performed by varying the adsorbate concentration from 20 to 500 mg L^−1^, and they were conducted for 24 h with an adsorbent dose of 2 g L^−1^ at pH = 4.

### 4.5. Analysis of Adsorption Data

The adsorption capacity (*q*_t_ or *q*_e_ if equilibrium is reached, mg g^−1^) was calculated from the initial (*C*_0_, mg L^−1^) and final copper concentrations (*C*_t_ or *C*_e_, mg L^−1^, respectively), adsorbent mass (*m*, g), and solution volume (*V*, L), according to Equation (4).

(4) $$q=\frac{V\left(C_{0}-C\right)}{m}$$

The analysis of the kinetic data was achieved using the pseudo-first-order, pseudo-second-order, and Weber–Morris models. In kinetic data, time *t* is expressed in hours. The pseudo-first-order model [93], Equation (5), is equivalent to some diffusion models and has been reported as only being valid at longer adsorption times. In this equation, *k*_1_ is the first-order rate constant (h^−1^). The pseudo-second-order model [94], Equation (6), can be derived from fundamental kinetic equations relating to surface reactions’ mechanisms. The pseudo-second-order adsorption rate constant is *k*_2_ (g mg^−1^ h^−1^). Nevertheless, both models can fit to all kinds of datasets, and definitive conclusions regarding sorption mechanisms cannot be drawn from this analysis alone [47]. To further clarify on adsorption mechanisms, the model presented by Weber and Morris (Equation (7)), describing intraparticle diffusion, was fitted to the data. In this equation, *K*_*WM*_ (mg g^−1^ h^−0.5^) is the rate constant and depends on the diffusion coefficient; *E* (mg g^−1^) is a constant shown to be correlated to the boundary layer thickness [95,96]; and the uptake is proportional to the square root of time [97]. In the kinetic models used, the equilibrium uptake or adsorption capacity, *q*_e_, is a model parameter.

(5) $$q_{t}=q_{e}\left(1-{\;e}^{-k_{1}t}\right),$$

(6) $$q_{t}=\;\frac{q_{e}^{2}k_{2}t}{q_{e}k_{2}t+1\;},$$

(7) $$q_{t}=k_{WM}t^{0.5}+E$$

The equilibrium data were interpreted by fitting the Langmuir, Freundlich, and Hill isotherms to the dataset. The Langmuir isotherm [98] (Equation (8)) considers monolayer adsorption at active sites, of homogenous surfaces that are identical and equivalent [99,100]. In this equation, *K*_L_ (L mg^−1^) is the Langmuir constant, and *q*_m_ (mg g^−1^) is the maximum adsorption capacity. The Freundlich model [101], Equation (9), describes adsorption on heterogeneous surfaces [99]. It has two parameters: the Freundlich constant, *K*_F_ ((mg g^−1^) (L mg^−1^)^1/n^_F_), which provides the relative adsorption capacity of the adsorbent, and the heterogeneity factor, 1/*n*_F_, which decreases with increasing heterogeneity. For the Hill equation (Equation (10)), *K*_H_ (mg L^−1^) is the Hill constant and *n_H_* is the Hill cooperativity factor.

(8) $$q_{e}=\frac{q_{m}K_{L}C_{e}}{1+K_{L}C_{e}}$$

(9) $$q_{e}=K_{F}{C_{e}}^{\frac{1}{n_{F}}},$$

(10) $$q_{e}=\frac{q_{m}C_{e}^{nH}}{K_{H}^{nH}+C_{e}^{nH}}$$

The nonlinear models were fitted using nonlinear regression with the Levenberg–Marquardt algorithm, and their quality was assessed using Akaike and Bayesian information criteria (AIC and BIC, respectively) [102]. The Weber–Morris equation is a linear model; hence, it was fitted using linear least squares, and its goodness of fit was assessed using the coefficient of determination.

## Figures and Tables

**Table 1:** Physical/structural properties of the chitosan–silica aerogel.

Bulk Density (kg m^−3^)	Skeletal Density (kg m^−3^)	Porosity (%)	*S*_BET_ (m^2^ g^−1^)	*V*_pore_ (cm^3^ g^−1^)	*D*_pore_ (µm)
56 ± 7	1258 ± 33	95.5 ± 0.6	2.05 ± 0.04	17 ± 2	33 ± 4

**Table 2:** CHNS composition of the chitosan–silica aerogel.

wt% C	wt% H	wt% N	wt% S
29.8 ± 0.2	4.62 ± 0.13	3.48 ± 0.03	2.92 ± 0.32

**Table 3:** Parameters of the kinetic models.

Pseudo-Second-Order Model				Pseudo-First-Order Model				Weber–Morris Model		
*q*_e_/(mg g^−1^)	*k*_2_/(g mg^−1^ h^−1^)	AIC	BIC	*q*_e_/(mg g^−1^)	*k*_1_/(h^−1^)	AIC	BIC	*k*_WM_/(mg g^−1^ h^−0.5^)	*E*/(mg g^−1^)	*R* ^2^
32.55 ± 0.08	0.79 ± 0.02	−19	−27	30.9 ± 0.6	14 ± 1	14	6	10 ± 3	22 ± 2	0.80

**Table 4:** Parameters of the isotherm models.

Langmuir Model				Freundlich Model				Hill Model				
*q*_m_ /(mg g^−1^)	*K*_L_ × 10^3^ /(L mg^−1^)	AIC	BIC	*1/n*_F_	*K*_F_/(mg g^−1^ (L/mg)^1/n^)	AIC	BIC	*q*_m_ /(mg g^−1^)	*K*_H_ /(mg L^−1^)	*n*_H_	AIC	BIC
39 ± 7	31 ± 19	39	31	0.3 ± 0.1	7 ± 5	42	34	32 ± 2	26 ± 2	8 ± 6	41	21

**Table 5:** Maximum adsorption capacity (*q*_max_) and adsorption equilibrium time (*t*_e_) reported for copper(II) removal using different adsorbents in the literature.

Adsorbent	*q*_max_/(mg g^−1^)	*t*_e_/min	Reference
*Chitosan–silica composite aerogel*	*39*	*<60*	*This study*
Chitosan-modified silica aerogel	34	120	[20]
Chitosan/waste glass (60 wt%) composite	36	30	[56]
Chitosan–nanoSiO_2_ nanocomposite	8	180	[57]
Chitosan/silica Cu(II)-imprinted microsphere	33	–	[58]
Chitosan/silica gel composite	1.3	120	[59]
Chitosan/silica microspheres	53	360	[60]
Silica/chitosan membrane	47	<1440	[61]
Silica gel/chitosan composite	870	15	[62]
Magnetic chitosan-tripolyphosphate@silica-coated composite	73	200	[63]
Chitosan-grafted-acrylic acid and modified nanosilica hydrogel	795	120	[64]
*β*-cyclodextrin-grafted-carboxymethylchitosan-modified silica gel	9	120	[65]
Carboxymethylchitosan-functionalized colloidal silica particles	172	60	[66]
Carboxymethylchitosan@silica-coated magnetic nanoparticles	346	120	[67]
Si/Fe nanostructures/chitosan composite	49	60	[68]
Polyacrylamide-grafted-chitosan/silica-coated Fe_3_O_4_ nanoparticles	45	15	[69]
Fe_2_O_3_@SBA-15−chitosan−APTMS composite	107	10	[70]
Fe_3_O_4_@SiO_2_@chitosan magnetic nanoparticles	7	90	[71]
Chitosan-SiO_2_@TEuTTA fluorescent membrane	51	120	[72]
Chitosan–SiO_2_ composite	642	–	[73]
Chitosan–natural zeolite composite	604	–	[73]
Chitosan–glauconite composite	618	–	[73]
Chitosan–montmorillonite	596	–	[73]
Chitosan hydrogel	311	–	[73]
Macroporous chitosan membrane	26	900	[74]
Chitosan beads	33	50	[75]
Chitosan aerogel	35	–	[76]
Powdered chitosan	54	480	[55]
Reduced salicylaldehyde-modified chitosan polymer	78	840	[55]
Schiff base organically modified silica aerogel	14	180	[52]
Schiff base-functionalized silica aerogel	244	360	[77]
Amino propyl triethoxysilane-modified silica aerogel	48	1440	[43]
Mercapto-functionalized silica aerogel	51	120	[78]
Amino-mercapto-functionalized silica xerogel	140	30	[79]
Amine-modified silica aerogel	130	>1440	[46]
Amine-modified silica xerogel	124	160	[80]
Amidoxime-functionalized silica aerogel	534	120	[81]
Methyl acrylate-modified silica aerogel	219	60	[82]
Nano-silica aerogel gelatin	369	300	[83]
APTES and EDTA-modified silica aerogel	94	20	[84]
Hybrid surfactant-templated mesoporous silica material	25	3	[53]
4-phenylacetophynone 4-aminobenzoylhydrazone anchored silica gel	0.8	120	[85]
Polybenzoxazine aerogel	1.5	>2880	[54]
Activated carbon	24	–	[50]
Acidified multi-walled carbon nanotubes	25	–	[51]
Fe_3_O_4_/talc nanocomposite	21	2	[86]
*Sida hermaphrodita* biochar	33	240	[87]
Low-cost, unmodified biomaterials/waste (e.g., leaves, peels, shells, straws, pulps)	2–35	5–360	[88,89,90,91,92]

## Data Availability

All data and materials are available on request from the corresponding author. The data are not publicly available due to ongoing researches using a part of the data.

## Supplementary Materials

The following supporting information can be downloaded at https://www.mdpi.com/article/10.3390/gels10030192/s1. Figures S1–S3 can be found in supporting information: Figure S1. SEM image (a) of the copper-loaded chitosan-silica aerogel with three regions highlighted (i), (ii) and (iii); EDX spectrum of each region (i), (ii) and (iii), showing the main components of the aerogel after Cu(II) adsorption; Figure S2. Microstructure of the copper-loaded chitosan-silica aerogel at 2.50k× obtained by backscattered electron SEM imaging; Figure S3. EDX spectrum of the neat chitosan-silica aerogel.
