# Supplementary material for: Chitosan–Silica Composite Aerogel for the Adsorption of Cupric Ions
Source: Gels. 2024 Mar 11;10(3):192. doi: 10.3390/gels10030192 (PMC10969789; doi:10.3390/gels10030192)
Supplement: Supplementary file 1 [file gels-10-00192-s001.zip › gels-2882533-supplementary.pdf]

Supporting Information

Chitosan–Silica Composite Aerogel for the Adsorption of Cupric Ions

João P. Vareda [1], Pedro M. C. Matias [2], José A. Paixão [3], Dina Murtinho [2], Artur J. M. Valente [2,*] and Luisa Durães [1]

1 University of Coimbra, CERES, Department of Chemical Engineering, Rua Silvio Lima, 3030-790 Coimbra, Portugal; jvareda@eq.uc.pt (J.P.V.); luisa@eq.uc.pt (L.D.)

2 University of Coimbra, CQC-IMS, Department of Chemistry, Rua Larga, 3004-535 Coimbra, Portugal; petermatias1998@gmail.com (P.M.C.M.); dmurtinho@ci.uc.pt (D.M.)

3 University of Coimbra, CFisUC, Department of Physics, Rua Larga, 3004-516 Coimbra, Portugal; jap@fis.uc.pt

* Correspondence: avalente@ci.uc.pt

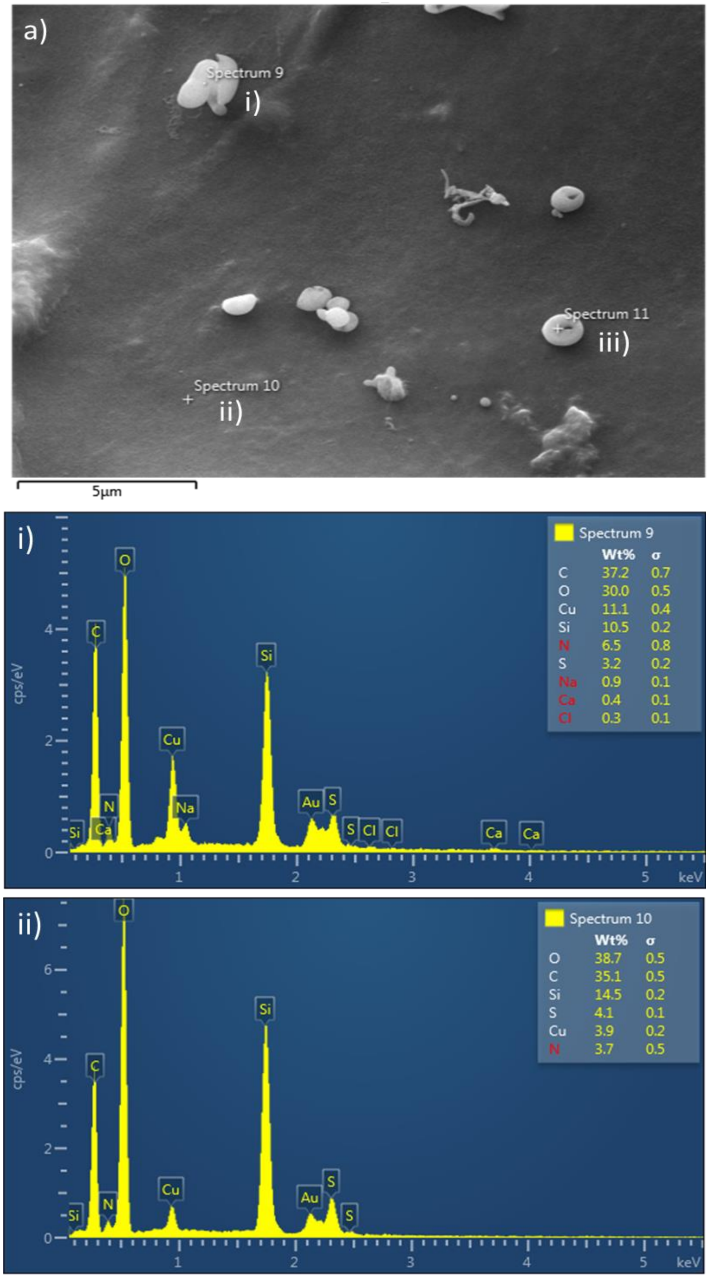

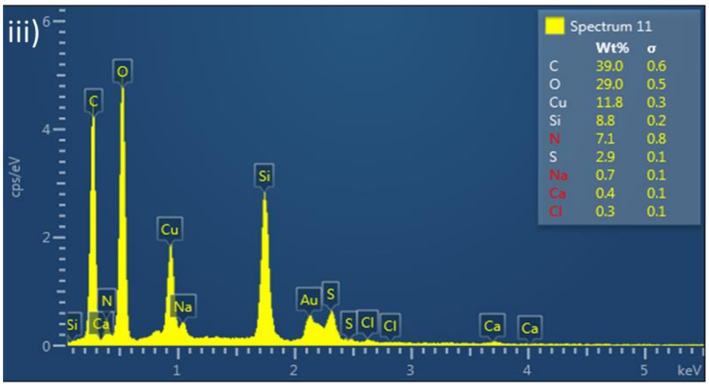

Figure S1. SEM image (a) of the copper-loaded chitosan-silica aerogel with three regions highlighted (i), ii) and iii)); EDX spectrum of each region (i), ii) and iii)), showing the main components of the aerogel after Cu(II) adsorption.

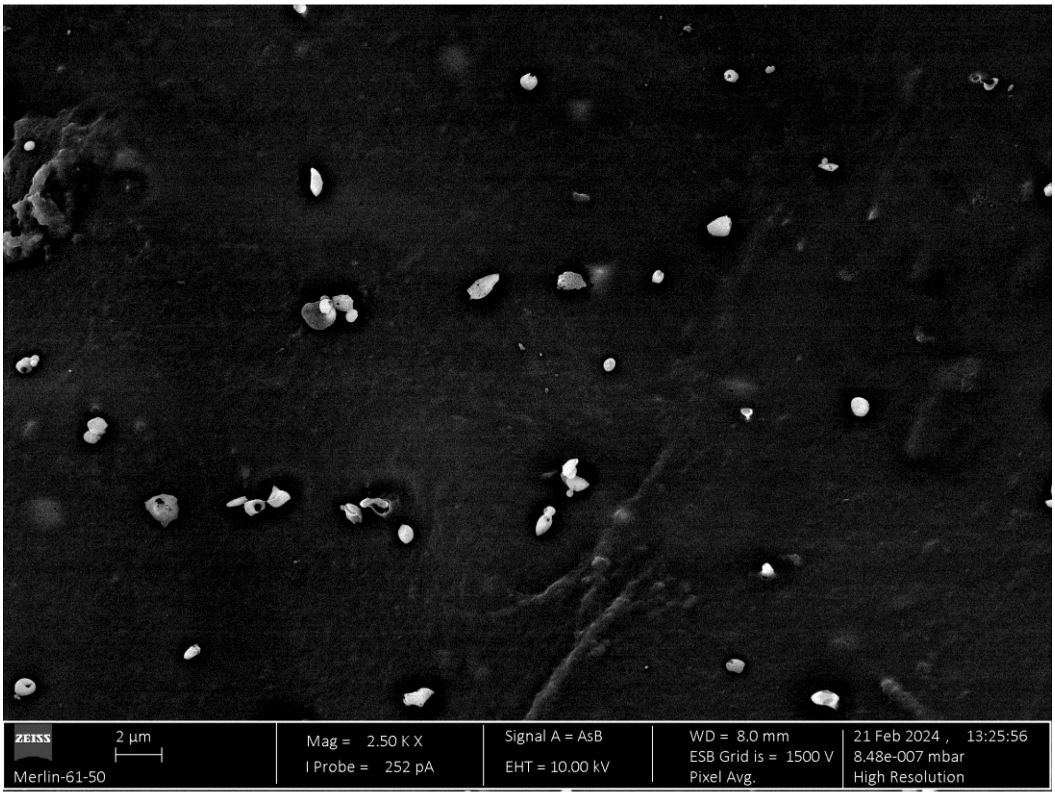

Figure S2. Microstructure of the copper-loaded chitosan-silica aerogel at 2.50k× obtained by backscattered electron SEM imaging.

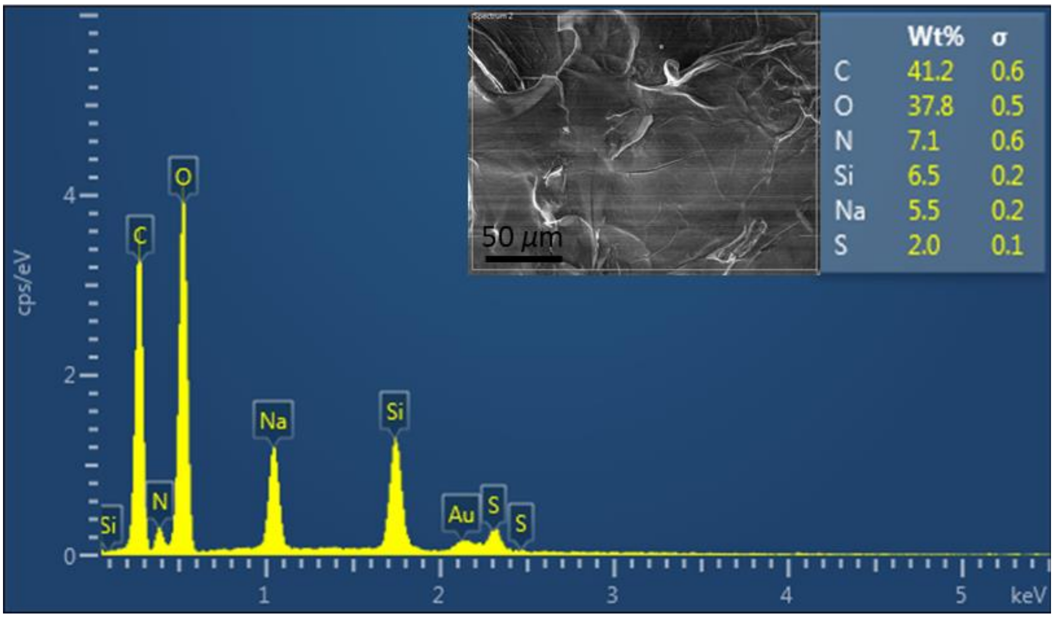

Figure S3. EDX spectrum of the neat chitosan-silica aerogel.
